# Dipole Source Localization of Mouse Electroencephalogram Using the Fieldtrip Toolbox

**DOI:** 10.1371/journal.pone.0079442

**Published:** 2013-11-14

**Authors:** Chungki Lee, Robert Oostenveld, Soo Hyun Lee, Lae Hyun Kim, Hokun Sung, Jee Hyun Choi

**Affiliations:** 1 Center for Bionics, Biomedical Research Institute, Korea Institute of Science and Technology, Seoul, South Korea; 2 Donders Institute for Brain, Cognition and Behaviour, Centre for Cognitive Neuroimaging, Radboud University, Nijmegen, The Netherlands; 3 Center for Neuroscience, Korea Institute of Science and Technology, Seoul, South Korea; 4 Department of Physics, Pohang University of Science and Technology, Pohang, South Korea; 5 Nano Process Division, Korea Advanced Nano Fab Center, Suwon, Gyeonggi, South Korea; 6 Department of Neuroscience, University of Science and Technology, Daejon, South Korea; City of Hope, United States of America

## Abstract

The mouse model is an important research tool in neurosciences to examine brain function and diseases with genetic perturbation in different brain regions. However, the limited techniques to map activated brain regions under specific experimental manipulations has been a drawback of the mouse model compared to human functional brain mapping. Here, we present a functional brain mapping method for fast and robust *in vivo* brain mapping of the mouse brain. The method is based on the acquisition of high density electroencephalography (EEG) with a microarray and EEG source estimation to localize the electrophysiological origins. We adapted the Fieldtrip toolbox for the source estimation, taking advantage of its software openness and flexibility in modeling the EEG volume conduction. Three source estimation techniques were compared: Distribution source modeling with minimum-norm estimation (MNE), scanning with multiple signal classification (MUSIC), and single-dipole fitting. Known sources to evaluate the performance of the localization methods were provided using optogenetic tools. The accuracy was quantified based on the receiver operating characteristic (ROC) analysis. The mean detection accuracy was high, with a false positive rate less than 1.3% and 7% at the sensitivity of 90% plotted with the MNE and MUSIC algorithms, respectively. The mean center-to-center distance was less than 1.2 mm in single dipole fitting algorithm. Mouse microarray EEG source localization using microarray allows a reliable method for functional brain mapping in awake mouse opening an access to cross-species study with human brain.

## Introduction

The mouse model is prevalent as a research tool for neurosciences in both normal and diseased models. With advances in genetic engineering, *in vivo* electrophysiological recordings such as electroencephalography (EEG) or local field potentials have gained traction as a meaningful approach to identify the disease phenotypes of gene modifications in terms of neural oscillation or evoked responses. However, due to the small size of mouse brain (approximately 1 cm^3^), no functional brain mapping equivalent to human neuroimaging is present. Recently, Mégevand *et al.* proposed a spatial mapping technique for modeling large-scale neuronal networks in mouse brains with a cluster of arranged electrodes, and successfully mapped cortical event-related potentials [1]. White *et al.* demonstrated an alternative method based on optical intrinsic signal imaging enabling the imaging of functional connectivity of mouse brain through skull [2]. However, while these advances have initiated ways to relate the human neuroscience and the transgenic mouse model, the experimental restriction requiring anesthesia is still an obstruction for placing the animal in the comparable conditions as human subjects during the acquisition of cortical activity related to experimental tasks.

With advance in signal processing, accurate estimation of the location of the generators and their sensitivity has been achieved by source localization of EEG in human brain [3]–[6]. Compared with other imaging modalities such as functional magnetic imaging or positron emission tomography, EEG has high temporal resolution presenting a solution to trace the signal transfer within different neuronal groups. Besides, EEG directly reflects the electrical current dipoles generated by synchronous depolarization or hyperpolarization of neuronal groups. The source localization of EEG consists of a forward problem to construct a realistic human head model and an inverse problem to infer the location of dipole from the potential distribution over the head [7], [8]. As the inverse problem is ill-posed problem, no unique solution exists, however current trends in adding *a priori* information or constraints to the problem enhanced the accuracy significantly [9]. Besides, EEG source localization has been validated using simultaneous invasive recording such as intracranial EEG [10] and lesion studies [11]. In the last ten years, EEG source localization has been widely used in clinical neurology (*e.g.*, neurology, psychiatry, and psychopharmacology) [12]–[14] as well as cognitive neuroscience (*e.g.*, attention, affective neuroscience) fields [15], [16]. Nonetheless, the innate limitation as ill-posed problems requires examination using invasive or lesion studies to receive a general credibility of the source estimation results.

In this paper, we describe a source localization method for mouse microarray EEG, which in turn delivers functional brain mapping in the mouse model that allows for cross-species comparison with the human brain. This is advantageous in the perspective of interrogation of neural circuits and imaging the concomitantly activated brain regions. To acquire the smooth potential map of mouse brain, we adapted the recently developed microarray based on polyimide substrate [17]. The polyimide-based microarray consists of 32 or 40 electrical contacts and a built-in connector to overcome the size problem of mouse brain, which has been a general hindrance for acquiring high density EEG For the forward problem, we constructed a boundary element model (BEM) based on magnetic resonance imaging (MRI) using conventionally available software. With the purpose of making the method reproducible to others, we implemented the source localization algorithm based on FieldTrip, an open source MATLAB toolbox [18]. Since the standard released version of FieldTrip assumes human head size dimensions in the source reconstruction, we adapted the algorithms to accommodate the mouse head size. Three source estimation techniques were used: Distribution source modeling with minimum-norm estimation (MNE), scanning with multiple signal classification (MNE), and single-dipole fitting (SDF). We validated the accuracy of the source estimation with known sources that were optogenetically stimulated *in vivo* in three different cortex of primary motor, primary somatosensory, and visual cortex at two different depths corresponding to cortical layer IV and VI.

## Materials and Methods

### 1. High Density EEG Microarray

The 40-channel microarrays were fabricated using a well-established nanofabrication process using polyimide substrate. The electrical contacts, the connection lines, and the interconnection pads were made of 300 nm-thick platinum deposited by a sputtering method on a spin-coated polyimide substrate (PIX-1400, HD MicroSystems, Japan). After patterning the metal layers using a photolithography process, a second 7-µm-thick layer of polyimide was spin-coated on top of the structure. The electrical contacts and the interconnection pads were then exposed by selective reactive-ion etching of the polyimide layer. Two connectors with 20 pins on each side (DF16B-40DP-0.5V, Hirose Electric Company, Ltd., Yokohama, Japan) were attached to the interconnection pads using soldering method to provide an interface to the recording equipment. All the nanofabrication process was conducted in Korea Advance Nano Fab Center (Suwon, South Korea).

The arrangement of the electrodes was determined to cover the most of the exposed skull and measure the surface potential with 500 um diameter electrode in a uniform way, where the diameter was determined based on the signal to noise ratio of EEG (Figure 1A in [17]). We sampled 8 weeks old mice to determine the dimension of the exposed skull. The extension to the temporal lobe was limited due to the inseparable connective tissue to the skull. The cortical map underneath the microarray is shown in the Figure S1.

**Figure 1:**
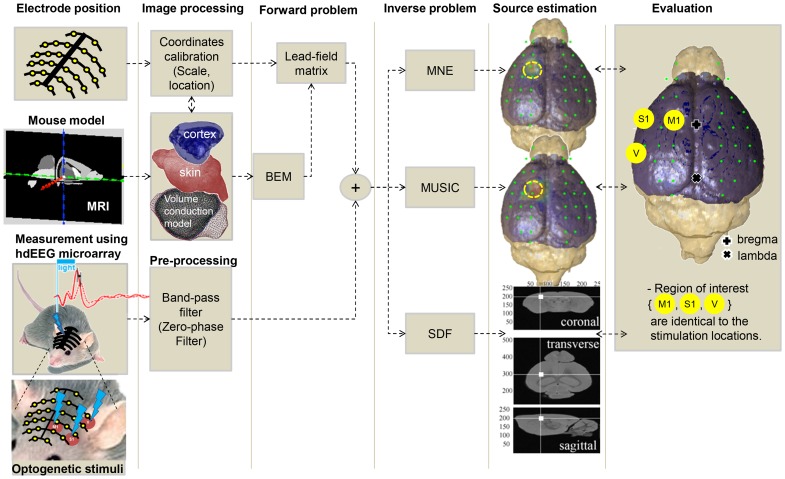
The Procedure of dipole source localization using mouse EEG. By nanofabrication of bifurcated and soft multi-channel microelectrode mounted on the skull surface, mouse EEG can be acquired in combination with optogenetic stimulation. Standard signal processing can be applied to the raw EEG signals (e.g. 60 Hz notch filter, 0.1 Hz high-pass filter, 50 Hz low-pass filter). Anatomical structures can be obtained from mouse MRI. To extract volume compartments we used commercial software (Curry 7, NeuroScan, Inc., Herndon, VA). The volume conduction model consists of brain and skull compartments and was generated with the boundary element model. Three different inverse solutions for estimating the dipole distribution were implemented and evaluated. To assess, we use three regions of interest at the stimulation location (M1, S1, and V).

### 2. High Density EEG Recording during Optogenetic Stimulation

All the mice used in this research were treated according to the Act 1992 of the Korea Lab Animal Care Regulations and associated guidelines. All the surgical and the experimental protocols for optogenetic brain stimulation of anesthetized mice were approved by the Institutional Animal Care and Use Committee in Korea Institute of Science and Technology, following Act 1992 of the Korea Lab Animal Care Regulations and associated guidelines (AP number: 2013-04-032).

We used Thy1-ChR2-EYFP transgenic mice (30–35 g in weight; 12–17 weeks; male). For *in vivo* recording, animals were anesthetized with intraperitoneal injection of ketamine/xylazine cocktail (120/6 mg/kg, respectively) and placed on the stereotaxic apparatus (David Kopf Instruments, Tujunga, CA, USA) with bregma and lambda points in the same horizontal plane. The scalp midline was incised to expose the skull. After removing debris on the skull with tap water soaked cotton balls, the microarray was aligned on the line between bregma and lambda. After fixing the microarray, holes of half a millimeter diameter were made using dental drill (model number: MARATHON-3, Saeyang Microtech, KOREA, drill size: 0.5 mm) in the skull for the optogenetic stimulation (Figure S1 (C)). The mice were fixed on a stereotaxic frame during recording. The detailed procedure for surgery is depicted in Lee *et al.* [19].

The mice were fixed on a stereotaxic frame during recording. For optogenetic stimulation, we used a semiconductor laser (USA & BCL-040-445; 445 nm wavelength and 40 mW/mm^2^ maximum output power; CrystaLaser LLC., Reno, NV, USA) that was gated using a pulse generator (575 digital delay, Berkeley Nucleonics Corp., Berkeley, CA, USA). Blue light from the laser was guided to the brain using an optic fiber with clad/core diameters of 125 µm and 3.4 µm, respectively (P1-405A-FC-5; Thorlabs Inc., Newton, NJ, USA). The light intensity from the tip of optical fiber was approximately 2 mW/mm^2^ measured by integrating sphere coupled to spectrometer (BLUE-Wave-VIS2/IC2/IRRAD-CAL, Stellar-Net Inc., Tampa, FL, USA). A pulse train with a 20 ms pulse width at 1 Hz was delivered in three different cortical regions (primary motor, primary somatosensory, and visual cortex marked as M1, S1, and V in Figure 1, respectively) at two different depths (500 and 800 µm ventral from dura). Each pulse train lasts 100 sec and at least 2 min of no stimulation was given after relocation of the optical fiber. The slow wave activity of EEG was monitored during EEG recording to administer additional Ketamine (40 mg/kg, i.p.) when the disruption of slow wave activity is monitored. The total time on stereotaxic instrument was approximately 1 hour.

After allocating two electrical contacts in the most posterior region as reference and ground electrodes, 38 active channel EEGs were recorded with a SynAmp^2^ amplifier (Neuroscan Inc., Herndon, VA), digitized with a 1 kHz sampling rate, and band-pass filtered from 0.1 to 100 Hz. Prior to recording, the impedance of the electrodes in the microarray was measured by using impedance meter embedded in SynAmp2 (test frequency of 30 Hz). Electrodes with an impedance greater than 300 kΩ (test frequency at 30 Hz) were excluded. Prior to topographical interpolation of the EEG for visualization, each channel time series was divided by a normalization factor that was defined by the average power of the range, 130 to 170 Hz within a quiescent moment of the animal.

### 3. Volume Conduction Model and Boundary Element Method (BEM)

The volume model was extracted from an MRI that was previously used in *in vitro* studies, and that was downloaded from the open database of the Magnetic Resonance Microimaging Neurological Atlas Group (http://brainatlas.mbi.ufl.edu/Database/). These were MRIs of brain without scalp and skull from four male, 12-week-old mice [20], [21]. Co-registration and Segmentation processes were performed using Curry software (version 7, Neuroscan Inc., Herndon, VA). Prior to segmentation, we scale up the mouse brain by tenfold along with the conductivity parameter to handle the *mm* resolution of the software. The threshold for segmenting the cortex from the MRI imaging was manually determined. To co-register the extracranial electrodes with the MRI coordinates, the following steps are carried out: First, we obtained the coordinates for the anatomical landmarks (nasion, preauricular left point, and preauricular right point) and the anterior and posterior commissures (AC and PC) in the MRI. Then we aligned the AC-PC to a horizontal line, and then manually designated the coordinates of border with respect to the AC (i.e., anterior, superior, inferior, left, and right borderes) and PC (i.e., posterior border) with the help of the *set parameter* menu in Curry software, then the coordinate of bregma point was estimated. Lastly, the electrodes are co-registered by adding the original coordinate measured with respect to the bregma point.

To overcome the individual difference of *in vivo* measured mouse and *in vitro* MRI model, we used the ratio of the length between bregma and lambda as a correction factor. The distance between bregma and lambda was measured and subsequently divided by 4.21 mm, the published average distance between lambda and bregma for C57BL/6J mice [22], and then subsequently multiplied by the distance between lambda and bregma in the MR imaging. We obtained the geometrical description of the BEM compartments using Curry software, which automatically segmented the relevant surfaces/compartment. To reduce the error in source localization due to anatomical difference between the measured brain and model, we aligned the midline of the microarray precisely along the line on the skull connecting bregma and lambda.

Following the extraction of the volume compartments, we used the FieldTrip software [18] for computing the BEM volume conduction model. This takes as input the triangulated surfaces that describe the boundaries and returns as output a volume conduction model that can be used to compute the leadfield matrices, which subsequently can be used in the inverse source estimation procedure. We assigned isotropic conductive properties for the compartments described by the triangular meshes, which consist of 5362 nodes in total (brain/skull: 2498/2864, respectively; mean node distances: 343/378 µm, respectively; conductivities: 0.33/0.0042 S/m, respectively) [23], [24]. An important consideration in the use of BEM is that its numerical accuracy depends on the size of the tessellation elements (triangle in the mesh) relative to the distance of the source, so that a finer tessellation is required when sources are close to surface [23].

### 4. Forward and Inverse Problems

To estimate the source parameters (location and/or strength), it is necessary to compute the leadfield matrix [25], [26]. The leadfield matrix describes the physical relations between the electrode potential and the modeled source activity. The leadfield matrix depends on the position of the model sources, the electrode position in the microarray, and on the geometrical and conductive characteristics of the volume conductor. The leadfield for the microarray was computed for 26510 dipoles placed at a regular grid location inside the brain compartment (i.e. the cortex).

To estimate the neuronal activation problem given the measured data (i.e. the inverse problem), we employed MNE, MUSIC, and SDF. The MNE is favored for analyzing evoked responses that involve wide-spread neuronal activation over time, resulting in the inverse solutions for the amplitude of a distributed model that discretizes the source space into locations on the cortical surface or in the brain volume using a large number of equivalent current dipoles. The MNE estimates the amplitude of all modeled source locations simultaneously with minimum overall energy [27]–[29].

The MUSIC is based on the singular value decomposition (SVD) method to identify the underlying components in the time series data that projected into an estimated signal subspace. The *p*-dimensional signal subspace is defined as corresponding to that part of the column space of M whose corresponding singular values λ_1_, λ_2_, … λ_p_ lie above a noise floor. Writing the SVD of the data matrix as M = UΛV^T^, the first *p* left singular vectors of M define the signal subspace U_p_, *i.e.*, U_p_ is formed from the first *p* columns of U. Source estimations for each grid location are found as inner product between U_p_ and the leadfield matrix [30], [31]. In the MUSIC method the whole cortical grid is scanned, resulting in a distributed representation of the estimated source activity.

The SDF method performs a grid-search based on one dipole and identifies the dipole model parameter that minimize the error between simulated and measured voltage distribution [32]. The resulting equivalent dipole model is interpreted as the most probable explanation for the actual cortical activity. Because of its simplicity, it is best, however, to limit its use to cases where a single dominant source can be assumed. The single equivalent current dipole model is not appropriate to explain distributed cortical activity or activity at different places simultaneously.

### 5. Validation of Estimated Source

In the optogenetically evoked responses in EEG, we estimated the source model for the earliest appearing responses to exclude more wide-spread sequentially evoked response that are due to synaptic projection. A comparison is performed to validate the reconstructed source using the gold standard for the stimulated cortical activity, characterized by distance from the optical stimulation site. To objectively assess the relative performance of the MNE and MUSIC inverse methods, we constructed the receiver operating characteristic (ROC) curve to analyze the trade-off between sensitivity and specificity. In a ROC curve, the true positive rate (sensitivity) is plotted against the false positive rate (1-specificity) with varying decision threshold. Standard ROC analysis can be applied for medical imaging given knowledge of the ground truth (*e.g.*, the location of a lesion) [33]. In most of human EEG, the ground truth is not known, but in our case it is the ChR2-induced spiking. Since it is more realistic to use a volume of activity rather than a single point, we adapted the free-response ROC described by Darvas, *et al.* [34].

The radius of the known source is estimated to range from 0.4 to 0.8 mm according to the following steps: (i) in brain tissue, the light intensity of 10 mW/mm^2^ is decreased to 1 mW/mm^2^ at 1.4 mm [35]. (ii) In our preliminary studies with optrode (core-to-core distance is 150 µm), the minimum light intensity to evoke field potential was 0.5 mW/mm^2^ (data not shown) in Thy1-ChR2 mice. (iii) In Beer-Lambert's law, the attenuation of light can be described as *ln*(I/I_0_) = −*ε·c·d*, where *ε* is the specific extinction coefficient of the chromophore, *c* is the concentration of the chromophore, and *d* the distance between light entry and exit point. By combining (i) and (ii), the effective distance for inducing ChR2-induced spiking for 2 mW/mm^2^ is approximately 0.84 mm. Likewise, the distance emitting 1 mW/mm^2^ for 2 mW/mm^2^ is approximately 0.42 mm. Hence, the decision threshold for ROC ranged from 0.4 to 0.8 mm. The ROC curves were produced both for this lower and upper bounds for the spatial extent of the stimulated cortical activity, by varying the threshold for the estimated dipole source amplitude.

The performance of ROC was assessed by the area under curve (AUC). The AUC is widely recognized as index of detection accuracy in medical imaging [36]. The maximum value for AUC is 1.0 indicating a perfect discrimination of an activating piece of cortex from a non-activating one, whereas an AUC value of 0.5 indicates no discriminative value. In the ROC curve this is noted by a straight, diagonal line from the lower left corner to the upper right corner (equal trade-off between sensitivity and specificity) [37].

In the case of SDF, we calculated the Euclidean distance between the stimulation spot and the best-fitting dipole location to assess the performance of inverse solution.

## Results

The procedures for the functional brain mapping for mouse EEG based on dipole source estimation are summarized in Figure 1. Briefly, the boundary element model for the mouse head was constructed based on MRI and accordantly the leadfield matrix was computed. Three different algorithms for dipole source estimation were evaluated in the *in vivo* EEG signals and compared to the known locations of the sources in three different cortices and two different cortical layers. To visualize the estimated dipole sources, we showed the absolute value of the reconstructed activity at the first peak of the optically evoked responses. No threshold to eliminate the background activity was needed in case of optogenetically evoked responses.

**Figure 2:**
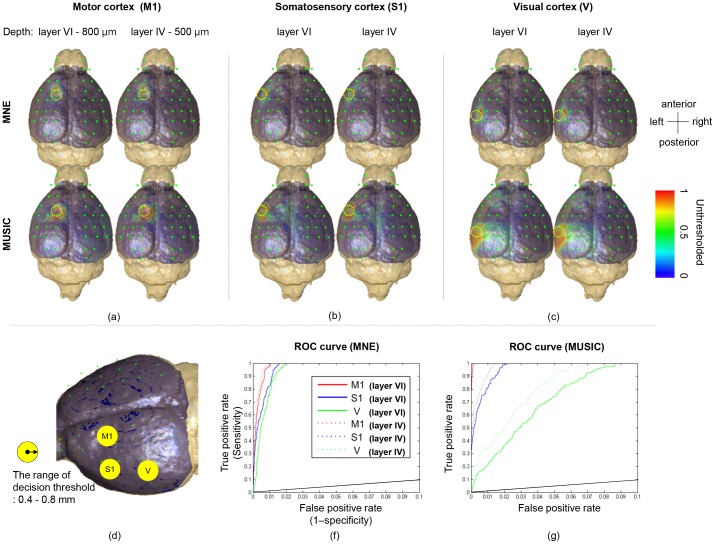
The results of source reconstruction for MNE and MUSIC. (a, b, c) reconstructed distributions with respect to inverse solution, source location, and depth show that estimated distribution with MNE produce as localized color at stimulated area, whereas MUSIC we easily observed that the reconstructed distribution is well-focalized by the MNE algorithm. The volume of estimated activated area is noted by yellow dotted circle, which is estimated to be ranged from 0.4 to 0.8 mm. The radius of the yellow circle is 0.6 mm. (d) Cortex volume and targeted areas (M1, S1, and V). The ROC curves for prediction of EEG source using MNE (f) and MUSIC algorithms (g). The black line is the diagonal line of no-discrimination.

Figures 2 illustrate the ability of MNE and MUSIC methods to localize differently located cortical sources in mouse brain. Visual inspection shows that the reconstructed source distributions are in good agreement with the stimulated region (yellow circle). Both MNE and MUSIC methods were able to localize the source, although the spatial extent of the source is slightly over-estimated. Stimulation of the medial cortex (M1) shows more focalized reconstruction than of the lateral cortices (S1, V). The comparison of the methods shows that MNE presented more focalized sources than MUSIC. An anterior-shift was observed in all the MNE reconstructions, where MUSIC showed more diffusive sources, but without shift.

The advantage of optogenetic stimulation is that the center and spatial extent of the true neural activation can be inferred from the tip location of the optical probe. The approximate range of the photon illumination is depicted in Figure 2(d) and marked by M1, S1 and, V.

We plotted the performances of MNE and MUSIC reconstruction methods as ROC curves for the radius of 0.4 and 0.8 mm, which is the lower and upper limit of the radius of the stimulated volume (used as the decision threshold for ROC curves). The ROC curves corresponding to the three locations (M1, S1, V) and two depths (layer IV and VI) are shown in Figure 2(f, g). For the varying thresholds of the MNE and MUSIC source reconstruction maps, the true positive rate (sensitivity) was obtained by calculating the ratio of above-threshold voxels within the stimulated volume; the false positive rate was obtained by calculating the and the ratio of above-threshold voxels within the whole brain space excluding the stimulated volume. Each ROC curve in Figure 2(f, g) rises rapidly towards a sensitivity of 100%. More specifically, in M1 stimulation, both MNE and MUSIC showed ROC curves close to an ideal ROC, where MNE shows a lightly higher false positive rate compared to MUSIC. In cases of S1 and V stimulations, the ROC curve in MNE increases faster than in MUSIC indicating an improvement in the detection accuracy of source estimation by using MNE for laterally located dipole sources. The false positive rate (i.e. 1-specificity) expressed a worse case of approximately 1.2% and 7% chances of a false positive per non-activating voxel with a true positive rate (sensitivity) exceeding 90% in MNE and MUSIC, respectively. Considering that the false positive rate does not exceed 1% with a 100% sensitivity in MNE estimation of M1 data, it is observed that MNE results in more reliable source estimation compared to MUSIC in case of single focused dipole in mouse model. In either case, the ROC curves demonstrate good ability to distinguish activated from non-activated brain tissue, compared to chance level detection which is marked by the diagonal black line.

For a quantitative evaluation, the AUC was calculated as decision accuracy index and summarized in Table 1. We found an excellent agreement between the known and estimated sources for all six locations. The AUC values of >0.99 in MNE and >0.97 in MUSIC demonstrates that the performance of source estimation for single focused activation was nearly perfect. Comparing the AUC values for two different cortical depths, the deeper layer, VI, presented slightly higher AUC values in all the cortex and methods, but not in a statistically significant way.

**Table 1:** Summary of AUC values for comparisons of detection accuracy of MNE *vs.* MUSIC algorithms in three cortical regions of M1, S1, and V, and in two cortical layers of IV and VI.

		Algorithms for inverse problem				
		Radius of known sources				
		*lbl*		*ubl*		
Stimulation location	Cortical layer	MNE	MUSIC	MNE	MUSIC	Average
M1	VI	0.9980	0.9996	0.9974	0.9998	**0.9987**
M1	IV	0.9982	0.9998	0.9979	0.9998	**0.9989**
S1	VI	0.9947	0.9990	0.9955	0.9956	**0.9962**
S1	IV	0.9948	0.9994	0.9956	0.9973	**0.9968**
V	VI	0.9924	0.9820	0.9936	0.9675	**0.9839**
V	IV	0.9924	0.9901	0.9936	0.9779	**0.9885**
**Average**		**0.9951**	**0.9950**	**0.9956**	**0.9897**	

The *lbl* and *ubl* are the lower bound limit and upper bound limit of the radius of known sources, which correspond to 0.4 and 0.8 mm, respectively.

**Figure 3:**
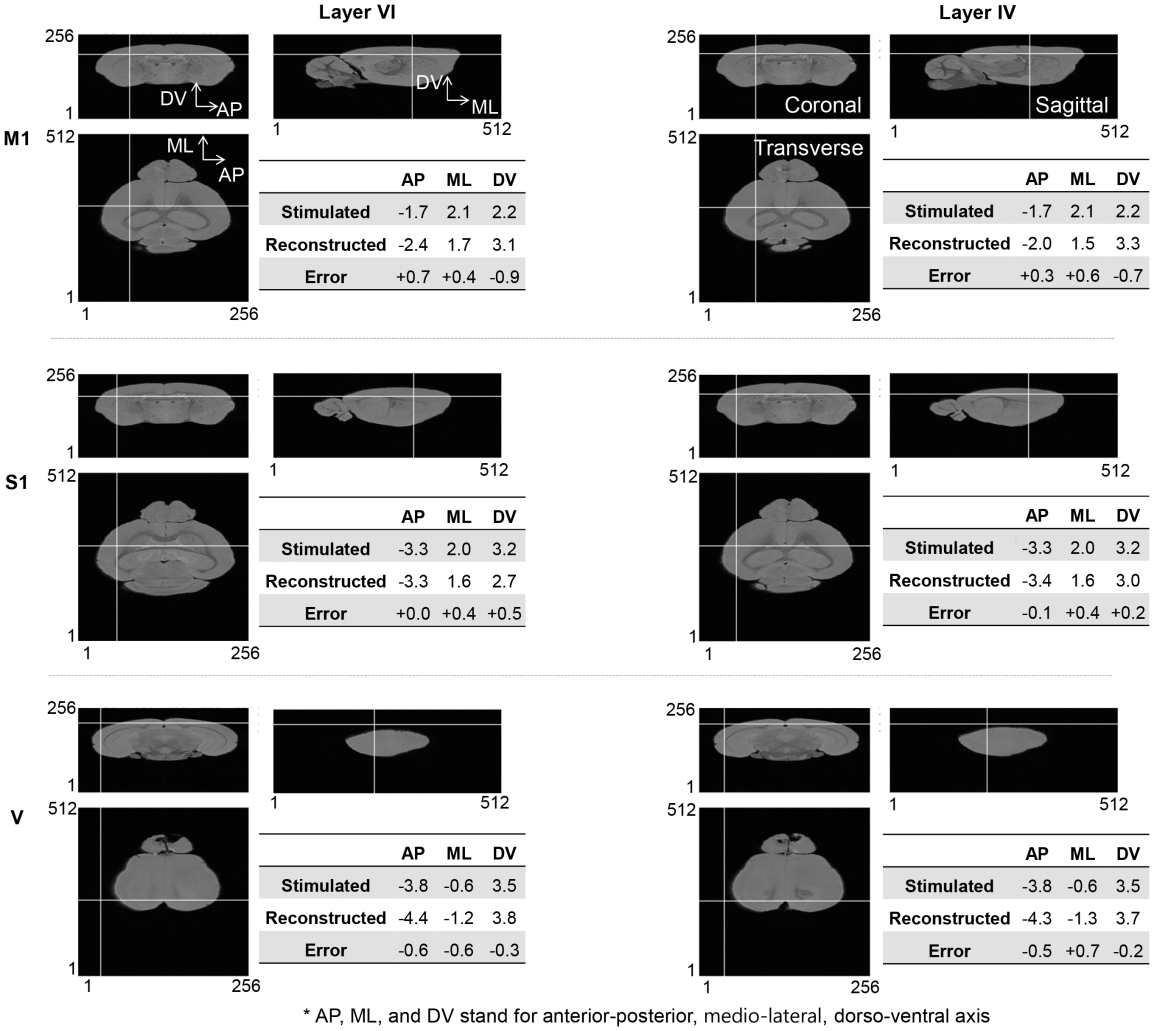
The results of single dipole fitting with respect to optogenetic stimulation at different locations (M1, S1, and V) and cortical depths (800 µm, layer VI and 500 µm, layer IV). The table shows are the center position of the stimulation cortex and the estimated dipole location. The positive signs for AP, ML, and DV errors mean that the SDF were biased to posterior, medial, and ventral direction, vice versa. The dominant single dipole was identified and visualized at the intersection of the overlaid horizontal and vertical lines on mouse brain MRI images. Orthogonal slices through the mouse MRI images are shown as transverse, sagittal, and coronal view, in each panel.

Figure 3 shows the estimated source location identified as best fitting the data by the SDF method, marked by the intersection point of the horizontal and vertical lines. To reduce the computation time, we used a sparser grid by skipping 2 grids in the original resolution of the head model. In this way, the computational time was reduced markedly from 150 to 10 s and the spatial resolution dropped by 70% but no apparent differences in estimated dipole source location between original and downscaled resolutions were found. We quantitatively evaluated the performance of SDF by calculating the Euclidian distance between the stimulation center and the dipole position in Table 2. The values of distance were higher in layer VI than in layer IV, implying that the accuracy of SDF is decreased for deeper sources. The SDF successfully reproduced the depth difference of layer VI and IV in case of M1 stimulation, however, the SDF failed to discriminate two cortical layers in cases of S1 and V stimulation in terms of position in the dorsal/ventral axis. In absolute position in dorsal/ventral axis, the deeper optogenetic stimuli (800 µm, layer VI) delivered the higher scoring indices compared to the shallower stimuli (500 µm, layer IV). Regardless of stimulation location, the reconstructed dipole position was shifted to the medial side. No systematic error was observed along the anterial/posterial axis. The distances between stimulation center and dipole position in each axis were less than 0.7, which is smaller than the estimated spatial extent of the ChR2 stimulation (see Method).

**Table 2:** Summary of Euclidean distance between the center of known source and the localization by SDF algorithms in three cortical regions of M1, S1, and V, and in two cortical layers of IV and VI.

		Axial bias (mm)			
Stimulation location	Cortical layer	AP	ML	DV	Euclidean distance (mm)
M1	VI	0.7	0.4	0.9	**1.2**
M1	IV	0.3	0.6	0.7	**1.0**
S1	VI	0.0	0.4	0.5	**0.6**
S1	IV	0.1	0.4	0.2	**0.4**
V	VI	0.6	0.6	0.3	**0.9**
V	IV	0.5	0.7	0.2	**0.9**

AP, ML, and DV are anterior-posterior, mediolateral, and dorso-ventral axis of brain, respectively. The axial bias is the absolute distance between the coordinates of stimulation and SDF centers in each axis.

## Discussion

We have presented a new method for functional brain mapping in the mouse model based on high-density EEG source localization methods. We demonstrate the ability to localize sources of optically evoked responses in the EEG by optogenetic stimulation. We applied three source localization techniques that all are able to detect the location of the sources underlying the hdEEG: MNE, MUSIC, and single dipole fitting. To increase the accessibility of this method, the functional mouse brain mapping method was implemented using FieldTrip, the open source Matlab software toolbox.

### 1. Validation of Source Localization using Optogenetic Stimulation

To quantitatively evaluate the validity and reliability of source localization, known cortical activity needs to be associated with spatially localized EEG generators. Several studies have been performed on validating the source localization from postoperative outcome in epilepsy patients [38], [39]. However, these validation methods suffer from multiple sources of epileptiform EEG activity potentially generated by subsequent postsynaptic activity. Conventional lesion biopsy techniques such as laser-based biopsy or stereotaxic-guided brain suction have been used in human brain [40], [41], but in these the lesion size are comparably large and the characteristic waveforms of the biopsied brain tissue were not characterized. The recently developed optogenetic tools excite neuronal tissue with genetically encoded ChR2 conferring millisecond precision [42]. Whereas the range of photon illumination for optogenetic excitation is relatively predictable using the optical properties of the tissue, it is difficult to predict the spatial range of the electrical dipole induced by neuronal spikes. So far, the relation between spikes and field potentials are known in case of synchronous responses to stimuli [43], [44]. However, in the case of scalp EEG, a recent study showed that the EEG reflects the dipole current of upper layers, basically the passive return sink via laminar analysis of slow wave activity [45]. Nonetheless, the largest dipole currents are positioned in the neighborhood of activating (hyperpolarized in this case) neurons. Therefore, the use of optogenetics to generate known sources is acceptable given the tolerance ranges of photon illumination, excitability of ChR2 expressed neurons, efficacy of ChR2 expression in Thy1, and inhomogeneous conductivity of extracellular fluid, whose order of magnitude is considered to be less than 1 mm.

### 2. Mouse Head Model for Forward Problem

We constructed a geometrical model of the mouse head using Curry 7 (Neuroscan, Inc., Herndon, VA) from available data in the Magnetic Resonance Microimaging Neurological Atlas Group [20], [21]. by segmenting brain tissues from T1-weighted magnetic resonance images. The layer of cerebral spinal fluid was neglected. Since the MRI data includes only the brain tissue, the skull was modeled by closed surfaces with known thicknesses of skull [46].

One of the major concerns of our head model is that two skull holes for eye sockets located at the lateral end of coronal suture were not considered. According to source localization study on the skull with a hole, the EEG is strongly distorted by the presence of a hole due to the current leakage especially when the sources near the border of the hole [47], [48]. The source nearest to the hole in our stimulation experiments was for the M1 stimulation, which is approximately 3 mm away from the boundary of the hole. The current leakage through the eye socket is expected to shift the source along the anterior-lateral side. We didn't particularly observe the anterior-lateral shift in case of M1 stimulation; however one should consider the possibility of current leakage through the eye socket hole. The cortical areas near the hole are the lateral part of primary and secondary motor cortex.

The individual head size difference was normalized by using the distance between bregma and lambda as a scaling parameter, which is a commonly accepted normalization method in stereotaxic study of rodents; however an inaccuracy may be present in the identification of the lambda point in the MR imaging due to absence of sutures; we estimate this inaccuracy and the resulting error to be less than 50 µm.

### 3. Source Localization of Optically Evoked Response in Mouse M1, S1, and V

Whereas validation metrics are suggested to quantify the performance of source localization algorithms [49], [50], no consensus is actually accepted [51]. Whereas we measured the geometric distance between estimated dipole source and the stimulation spot in case of SDF, we applied ROC analysis to specifically assess detection accuracy in cases of MNE and MUSIC. The sufficient condition of the application of the ROC analysis is the presence of a ground truth (golden standard), which is fulfilled with optogenetic stimulation. According to ROC analysis, MNE and MUSIC estimated the activated areas correctly with a low level of trade-off in exclusion of the non-activating ones. Considering AUC>0.8 as acceptable detection accuracy, the AUC>0.97 implies that the dipole source of single focalized activation can be detected exquisitely in mouse model. More precisely, MUSIC showed more accurate localizations in M1, whereas MNE showed more accurate localization in V. It has been reported that MNE yielded more precise and accurate source detection in case of focalized and superficial sources [52]. In our study, MNE studies showed generally better localized sources regardless of the stimulation location, but no difference was found between different cortical layers. On the other hand, MUSIC showed more extended source distribution estimates. In sum, MUSIC predicted the frontal and central sources better than MNE, and MNE showed better performance for the lateral sources compared. In either method, the sources in the layers IV and VI, which corresponds to afferent and efferent portions, respectively, were indistinguishable. In SDF, the layer selectivity was not successful. In each method, the dipole prediction in the cases of activation located underlying the electrode coverage yielded better performance.

## Conclusion

We have successfully implemented and demonstrated source localization reconstruction for mouse EEG using open source software, FieldTrip. Quantitative voxel-wise comparison and validation of source reconstruction for mouse EEG against the neuronal activation evoked by optical stimulation has produced accurate localization of different cortical areas. While further works remain to be done to further improve the layer selectivity, the separability of multiple sources, or the focalizability of lateral source, the microarray EEG based imaging has valuable potential for linking human brain mapping with the genetic/molecular/circuit perturbation in mouse models.

## Supporting Information

Figure S1 High density mouse EEG and mouse head model. (a) polyimide based microelectrode array (b) mouse with exposed skull after placement of microelectrode array (c) volume conduction model (skull layer: black mesh, cortical layer: red mesh), overlapped image with locations of microelectrode layout (black dots) and optogenetic stimulation (yellow dots) (d) functional-anatomical map of mouse cortex and arrangement of microelectrode array.
(TIF)
