# Supplementary figures and images for: Dipole Source Localization of Mouse Electroencephalogram Using the Fieldtrip Toolbox
Source: PLoS One. 2013 Nov 14;8(11):e79442. doi: 10.1371/journal.pone.0079442 (PMC3828402; doi:10.1371/journal.pone.0079442)

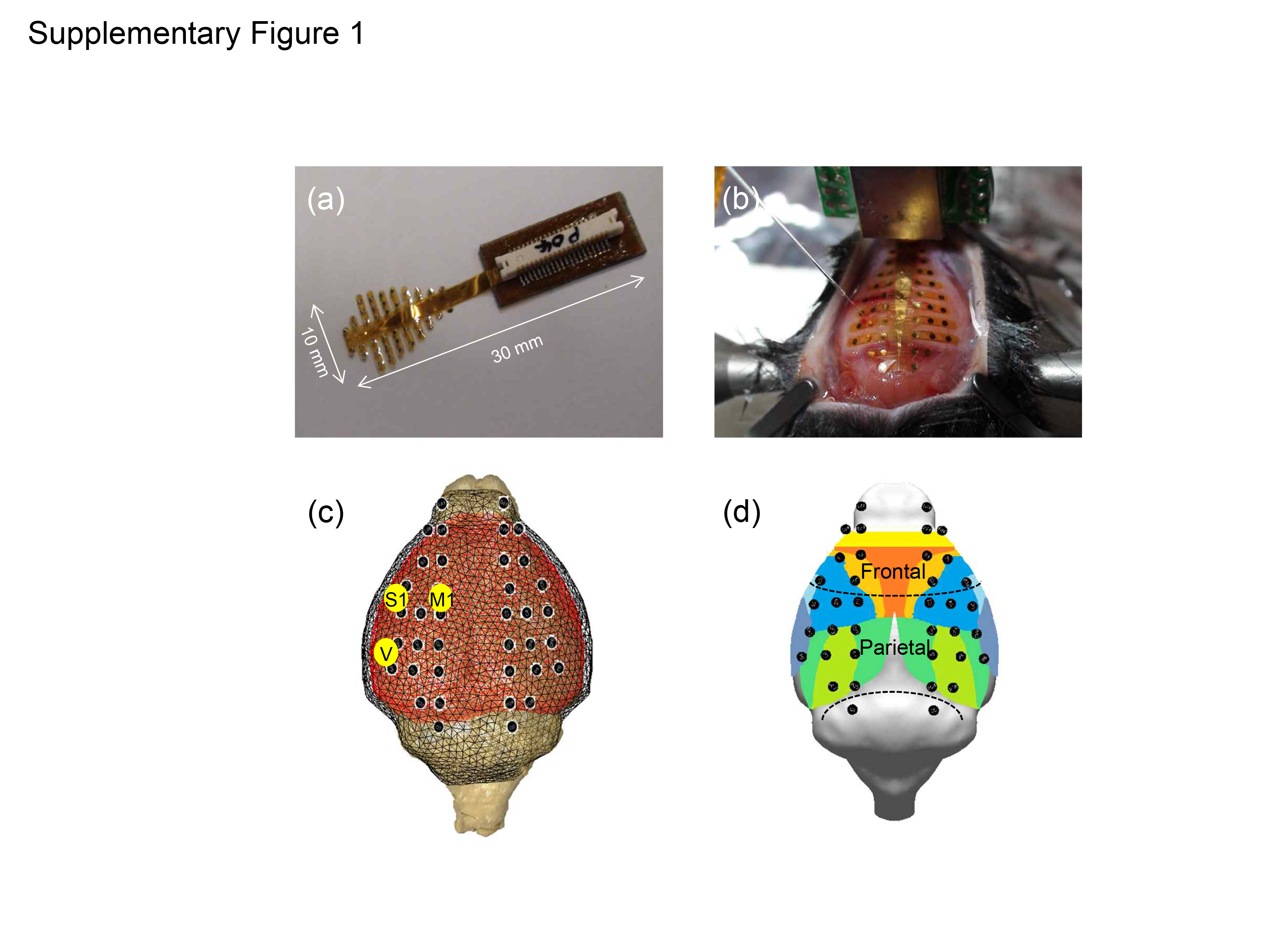

Supplement: Figure S1 — High density mouse EEG and mouse head model. (a) polyimide based microelectrode array (b) mouse with exposed skull after placement of microelectrode array (c) volume conduction model (skull layer: black mesh, cortical layer: red mesh), overlapped image with locations of microelectrode layout (black dots) and optogenetic stimulation (yellow dots) (d) functional-anatomical map of mouse cortex and arrangement of microelectrode array. (TIF) [file pone.0079442.s001.tif]
